# Supplementary material for: Validation of a Quantitative Proton Nuclear Magnetic Resonance Spectroscopic Screening Method for Coffee Quality and Authenticity (NMR Coffee Screener)
Source: Foods. 2020 Jan 4;9(1):47. doi: 10.3390/foods9010047 (PMC7023380; doi:10.3390/foods9010047)
Supplement: Supplementary file 1 [file foods-09-00047-s001.pdf]

Table S1. Raw results of method validation for coffee using a factorial experimental design

	Factor 1	Factor 2	Factor 3	Factor 4	Response 1	Response 2	Response 3	Response 4	Response 5
Run	Spiked concentration of all analytes (mg/kg)	NMR type	Extraction time (min)	Coffee type	Caffeine (mg/kg)	OMC (mg/kg)	Kahweol (mg/kg)	Furfuryl alcohol (mg/kg)	HMF (mg/kg)
1	7.5	Ascend	10	Decaf. arabica	10.5	17	162.5	n.d.	7
2	37.5	Ascend	10	Decaf. arabica	42.5	30	n.d.	13.5	35
3	75	Ascend	10	Decaf. arabica	83.5	60	324.5	64.5	85
4	187.5	Ascend	10	Decaf. arabica	203.5	190	n.d.	177.5	206
5	375	Ascend	10	Decaf. arabica	400.5	372	n.d.	361.5	412
6	750	Ascend	10	Decaf. arabica	786.5	767	260.5	720.5	813
7	1875	Ascend	10	Decaf. arabica	1924.5	1855	1726.5	1788.5	1854
8	3750	Ascend	10	Decaf. arabica	3827.5	3705	3103.5	3587.5	3691
9	5625	Ascend	10	Decaf. arabica	5765.5	5594	5073.5	5405.5	5581
10	7500	Ascend	10	Decaf. arabica	7517.5	7294	6419.5	7057.5	7282
11	7.5	Ascend	10	Canephora	n.d.	n.d.	n.d.	13	14.5
12	37.5	Ascend	10	Canephora	n.d.	n.d.	36.5	24	59.5
13	75	Ascend	10	Canephora	85.5	76	61.5	78	84.5
14	187.5	Ascend	10	Canephora	335.5	254	103.5	200	220.5
15	375	Ascend	10	Canephora	431.5	384	220.5	391	443.5
16	750	Ascend	10	Canephora	762.5	772	306.5	751	839.5
17	1875	Ascend	10	Canephora	1765.5	1809	1734.5	1836	1884.5
18	3750	Ascend	10	Canephora	3783.5	3691	3405.5	3667	3746.5
19	5625	Ascend	10	Canephora	5614.5	5544	5183.5	5446	5596.5
20	7500	Ascend	10	Canephora	7615.5	7437	6929.5	7302	7472.5
21	7.5	Ascend	20	Decaf. arabica	7	10.5	255.5	14	12.5
22	37.5	Ascend	20	Decaf. arabica	30	34.5	272.5	43	44.5
23	75	Ascend	20	Decaf. arabica	73	65.5	250.5	76	82.5
24	187.5	Ascend	20	Decaf. arabica	174	159.5	104.5	162	184.5
25	375	Ascend	20	Decaf. arabica	378	359.5	332.5	359	401.5
26	750	Ascend	20	Decaf. arabica	743	716.5	652.5	691	775.5
27	1875	Ascend	20	Decaf. arabica	1852	1801.5	2002.5	1741	1790.5
28	3750	Ascend	20	Decaf. arabica	3643	3561.5	3579.5	3450	3525.5
29	5625	Ascend	20	Decaf. arabica	5471	5336.5	5244.5	5184	5301.5
30	7500	Ascend	20	Decaf. arabica	7264	7088.5	6793.5	6869	7073.5
31	7.5	Ascend	20	Canephora	n.d.	n.d.	83	2.5	5
32	37.5	Ascend	20	Canephora	n.d.	n.d.	45	25.5	31
33	75	Ascend	20	Canephora	213.5	169	26	79.5	67
34	187.5	Ascend	20	Canephora	249.5	227	70	178.5	182
35	375	Ascend	20	Canephora	404.5	358	211	352.5	386
36	750	Ascend	20	Canephora	759.5	753	335	704.5	772
37	1875	Ascend	20	Canephora	1742.5	1749	1696	1723.5	1738
38	3750	Ascend	20	Canephora	3467.5	3477	3355	3451.5	3518
39	5625	Ascend	20	Canephora	5391.5	5320	5084	5200.5	5311
40	7500	Ascend	20	Canephora	6956.5	6969	6696	6896.5	7040
41	7.5	UltraShield	10	Decaf. arabica	16.5	n.d.	270.5	5.5	3
42	37.5	UltraShield	10	Decaf. arabica	49.5	22	n.d.	28.5	38
43	75	UltraShield	10	Decaf. arabica	89.5	79	169.5	75.5	73
44	187.5	UltraShield	10	Decaf. arabica	207.5	195	n.d.	181.5	198
45	375	UltraShield	10	Decaf. arabica	429.5	400	n.d.	381.5	439

46	750	UltraShield	10	Decaf. arabica	814.5	775	214.5	755.5	847
47	1875	UltraShield	10	Decaf. arabica	2034.5	1935	1897.5	1892.5	1935
48	3750	UltraShield	10	Decaf. arabica	4048.5	3916	3509.5	3801.5	3908
49	5625	UltraShield	10	Decaf. arabica	6040.5	5837	5357.5	5682.5	5836
50	7500	UltraShield	10	Decaf. arabica	7713.5	7457	6550.5	7265.5	7445
51	7.5	UltraShield	10	Canephora	n.d.	n.d.	n.d.	7	10.5
52	37.5	UltraShield	10	Canephora	n.d.	n.d.	n.d.	28	46.5
53	75	UltraShield	10	Canephora	3	39	64.5	77	87.5
54	187.5	UltraShield	10	Canephora	334	251	77.5	209	217.5
55	375	UltraShield	10	Canephora	379	370	255.5	418	439.5
56	750	UltraShield	10	Canephora	798	802	370.5	788	882.5
57	1875	UltraShield	10	Canephora	1892	1921	1803.5	1961	2007
58	3750	UltraShield	10	Canephora	4037	3947	3690.5	3890	3983
59	5625	UltraShield	10	Canephora	5974	5872	5492.5	5788	5928
60	7500	UltraShield	10	Canephora	8185	7993	7384.5	7790	7977
61	7.5	UltraShield	20	Decaf. arabica	18	n.d.	414	12.5	10
62	37.5	UltraShield	20	Decaf. arabica	39	17.5	243	37.5	32
63	75	UltraShield	20	Decaf. arabica	90	79.5	374	74.5	80
64	187.5	UltraShield	20	Decaf. arabica	193	163.5	102	180.5	197
65	375	UltraShield	20	Decaf. arabica	417	401.5	509	376.5	438
66	750	UltraShield	20	Decaf. arabica	811	765.5	871	750.5	834
67	1875	UltraShield	20	Decaf. arabica	1957	1887	2024	1833.5	1874
68	3750	UltraShield	20	Decaf. arabica	3893	3768	3733	3670.5	3758
69	5625	UltraShield	20	Decaf. arabica	5839	5664	5573	5493.5	5656
70	7500	UltraShield	20	Decaf. arabica	7769	7521	7172	7336.5	7539
71	7.5	UltraShield	20	Canephora	n.d.	n.d.	13.5	21	n.d.
72	37.5	UltraShield	20	Canephora	n.d.	4	n.d.	27	33.5
73	75	UltraShield	20	Canephora	237	180	65.5	81	77.5
74	187.5	UltraShield	20	Canephora	281	262	123.5	203	208.5
75	375	UltraShield	20	Canephora	421	377	190.5	380	409.5
76	750	UltraShield	20	Canephora	857	809	394.5	748	848.5
77	1875	UltraShield	20	Canephora	1974	1949	1835.5	1902	1931
78	3750	UltraShield	20	Canephora	3868	3841	3700.5	3789	3896
79	5625	UltraShield	20	Canephora	6024	5901	5622.5	5734	5866
80	7500	UltraShield	20	Canephora	7695	7662	7384.5	7581	7766
81	7.5	Ascend	10	Green coffee	n.d.	n.d.	n.d.	n.d.	9
82	37.5	Ascend	10	Green coffee	41	46	87	24	48
83	75	Ascend	10	Green coffee	75	78	34	57	80
84	187.5	Ascend	10	Green coffee	199	183	83	167	205
85	375	Ascend	10	Green coffee	371	359	n.d.	369	414
86	750	Ascend	10	Green coffee	776	726	112	714	809
87	1875	Ascend	10	Green coffee	1946	1819	1384	1816	1873
88	3750	Ascend	10	Green coffee	3765	3517	2964	3533	3560
89	5625	Ascend	10	Green coffee	3782	3668	2004	3649	3773
90	7500	Ascend	10	Green coffee	5111	4966	3165	4875	5060
91	7.5	Ascend	20	Green coffee	45.5	n.d.	194.5	11	14
92	37.5	Ascend	20	Green coffee	57.5	29	27.5	33	44
93	75	Ascend	20	Green coffee	n.d.	n.d.	n.d.	46	43
94	187.5	Ascend	20	Green coffee	223.5	198	104.5	176	211
95	375	Ascend	20	Green coffee	411.5	363	222.5	367	425
96	750	Ascend	20	Green coffee	830.5	730	384.5	728	848
97	1875	Ascend	20	Green coffee	1956.5	1829	1344.5	1822	1896
98	3750	Ascend	20	Green coffee	3805.5	3613	3454.5	3527	3650
99	5625	Ascend	20	Green coffee	3858.5	3738	1813.5	3731	3852

100	7500	Ascend	20	Green coffee	7916.5	7596	6483.5	7429	7708
101	7.5	UltraShield	10	Green coffee	n.d.	n.d.	n.d.	n.d.	6.5
102	37.5	UltraShield	10	Green coffee	n.d.	n.d.	1113.4	n.d.	n.d.
103	75	UltraShield	10	Green coffee	80	7.5	29	73.5	73.5
104	187.5	UltraShield	10	Green coffee	298.8	212.8	1433.3	191.5	197.3
105	375	UltraShield	10	Green coffee	n.d.	n.d.	899	n.d.	n.d.
106	750	UltraShield	10	Green coffee	809	723.5	148	740.5	855.5
107	1875	UltraShield	10	Green coffee	1996	1809.1	2977.2	1775.1	1781.5
108	3750	UltraShield	10	Green coffee	3035.7	2822.2	4075	2756.1	2825.1
109	5625	UltraShield	10	Green coffee	3217.6	3045.9	2941.5	3039.2	3112.8
110	7500	UltraShield	10	Green coffee	5369.3	5148.2	4154.7	5046.5	5221.8
111	7.5	UltraShield	20	Green coffee	31.5	n.d.	242.5	9.5	7.5
112	37.5	UltraShield	20	Green coffee	56.5	44	92.5	21.5	56.5
113	75	UltraShield	20	Green coffee	n.d.	1	n.d.	35.5	55.5
114	187.5	UltraShield	20	Green coffee	229.5	168	44.5	185.5	219.5
115	375	UltraShield	20	Green coffee	438.5	387	224.5	388.5	461.5
116	750	UltraShield	20	Green coffee	849.5	780	360.5	748.5	882.5
117	1875	UltraShield	20	Green coffee	2146.5	2003	1566.5	1992.5	2086
118	3750	UltraShield	20	Green coffee	4040.5	3825	3667.5	3734.5	3878
119	5625	UltraShield	20	Green coffee	4065.5	3955	1908.5	3924.5	4061
120	7500	UltraShield	20	Green coffee	8393.5	8102	6879.5	7840.5	8140

n.d., not detectable (response <0).

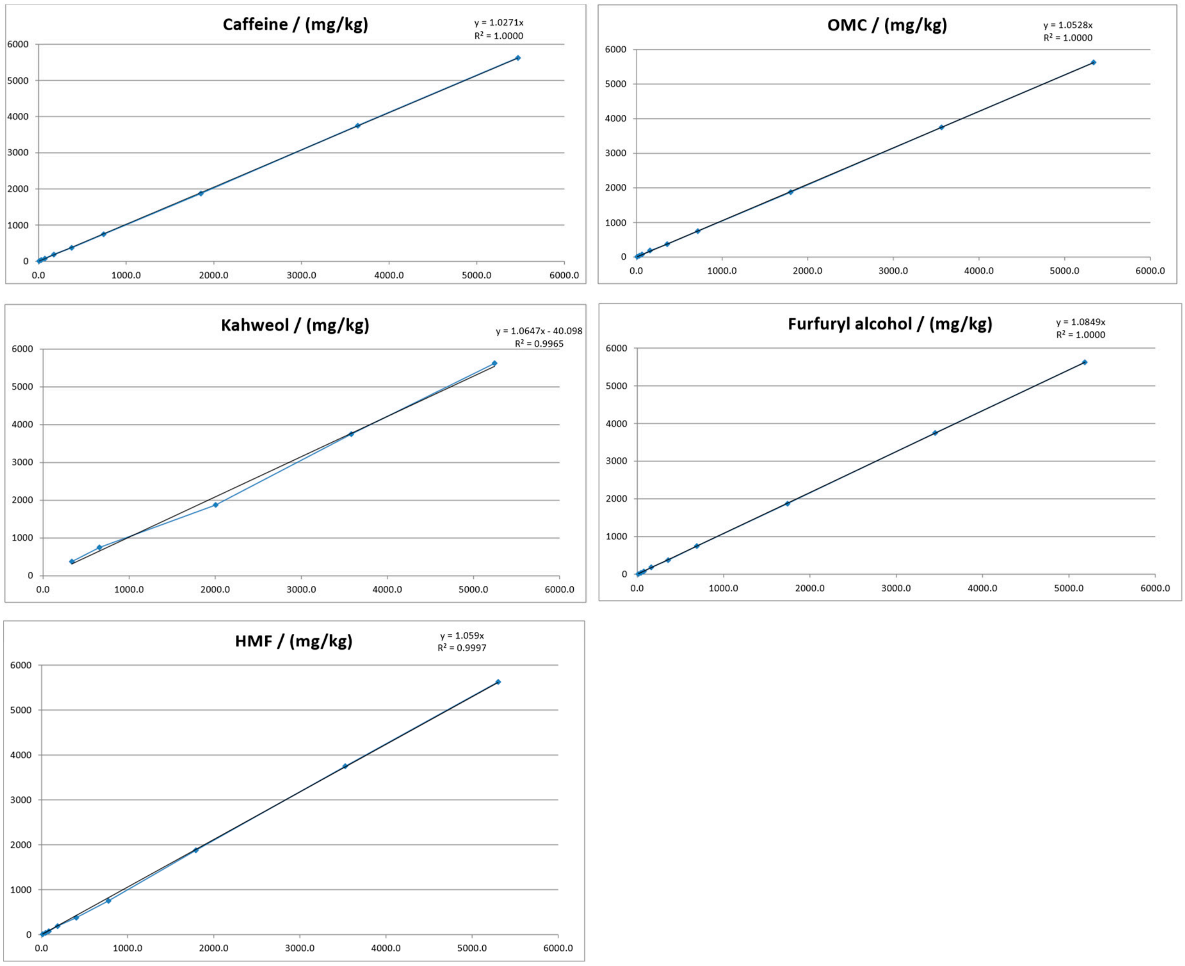

Figure S1. Linearity determination of target compounds.
